# Prevalence and Risk Factors for Hepatitis B Virus Infection in Roma and Non-Roma People in Slovakia

**DOI:** 10.3390/ijerph15051047

**Published:** 2018-05-22

**Authors:** Sylvia Drazilova, Martin Janicko, Pavol Kristian, Ivan Schreter, Monika Halanova, Ingrid Urbancikova, Andrea Madarasova-Geckova, Maria Marekova, Daniel Pella, Peter Jarcuska, HepaMeta Team

**Affiliations:** 1 Department of Internal Medicine, University Hospital Poprad, 058 01 Poprad, Slovakia; drazilova.s@nemocnicapp.sk; 2 1st Department of Internal Medicine, Faculty of Medicine, P.J. Safarik University and L. Pasteur University Hospital, Kosice, 040 11 Kosice, Slovakia; daniel.pella@upjs.sk (D.P.); peter.jarcuska@upjs.sk (P.J.); 3 Department of Infectious Diseases, Faculty of Medicine, P.J. Safarik University and L. Pasteur University Hospital, Kosice, 040 01 Kosice, Slovakia; kristian@unlp.sk (P.K.); ivan.schreter@upjs.sk (I.S.); 4 Department of Epidemiology, Faculty of Medicine, P.J. Safarik University, Kosice, 041 80 Kosice, Slovakia; monika.halanova@upjs.sk; 5 Department of Pediatrics, Faculty of Medicine and Children´s Faculty Hospital, P.J. Safarik University, Kosice, 040 11 Kosice, Slovakia; ingrid.urbancikova@upjs.sk; 6 Department of Health Psychology, Faculty of Medicine, P.J. Safarik University, Kosice, 040 11 Kosice, Slovakia; andrea.geckova@upjs.sk; 7 Department of Medical and Clinical Biochemistry, Faculty of Medicine, P.J. Safarik University, Kosice, 040 11 Kosice, Slovakia; maria.marekova@upjs.sk; 8 Faculty of Medicine, P.J. Safarik University, Kosice, 040 11 Kosice, Slovakia; peter.kolarcik@upjs.sk

**Keywords:** chronic hepatitis B, Roma population, prevalence, risk factors, anti HBc antibodies, HBV DNA

## Abstract

Prevalence of Hepatitis B is relatively low in developed European countries. However specific subpopulations may exist within each country with markedly different Hepatitis B burden. Roma minority is very numerous in Slovakia and their lifestyle is completely different to non-Roma population. The aim of this study is to map Hepatitis B prevalence in Roma and compare it to non-Roma population and to explore potential socio-economic and health related risk factors. Cross-sectional epidemiology study was performed in Slovakia that included randomly sampled Roma population and geographically corresponding random sampled non-Roma population. Comprehensive questionnaire about risk factors was administered and blood samples were drawn for Hepatitis B serology and virology tests. Altogether 855 participants were included. Global Hepatitis B surface Antigen (HBsAg) positivity rate was 7.7% (i.e., active Hepatitis B) and anti Hepatitis B core IgG antibody (antiHBcIgG) positivity rate was 34.6%. Roma population had significantly higher prevalence of Hepatitis B, both active chronic infection (12.4%; 95% Confidence Interval (CI) 9.58%–15.97% versus 2.8%; 95% CI 1.56%–4.91%; *p* < 0.0001) and antiHBcIgG positivity (52.8%; 95% CI 48.17%–57.44% versus 25.9%; 95% CI 12.56%–20.02%; *p* < 0.0001) Main risk factors for HBsAg positivity were Roma ethnicity, male sex and tattoo. Conclusion: There is a very high prevalence of Hepatitis B in Roma communities in Slovakia, with potential for grave medical consequences.

## 1. Introduction

Global estimated Hepatitis B surface Antigen (HBsAg) seroprevalence is 3.9% (95% uncertainty interval 3.4–4.6), corresponding to 291,992,000 (95% uncertainty interval 251,513,000–341,114,000) infections [1], which is a perceivable decrease from previously reported numbers but chronic Hepatitis B still presents a major global health burden. 

Hepatitis B can be transmitted vertically from mother to child or horizontally by infected blood or by sexual intercourse [2]. Almost a third of newly infected adults in the USA are infected via sexual intercourse, out of which 39% via heterosexual and 24% via homosexual contact [3]. Approximately three quarters of unvaccinated American homosexual men become infected by Hepatitis B virus after five years of regular sexual intercourse [4]. High risk of transmission by infected blood is associated with following medical and non-medical procedures:

- Blood products and whole blood transfusion
- Surgical and microsurgical procedures
- Haemodialysis
- Intravenous (i.v.) drug application
- Accidental puncture by infected needle
- Accidental contact with infected blood
- Tattoo or piercing cosmetic procedures
- Manicure or pedicure
- Shared use of razor or toothbrush [2,5].

Chronic Hepatitis B may progress to liver cirrhosis and hepatocellular cancer [5], that may occur in infected individual even without liver cirrhosis [6]. Decompensated liver cirrhosis and hepatocellular cancer frequently result in the fatal outcome. Annual liver related mortality in Hepatitis B infected patients is more than one million patients [7].

Hepatitis B vaccine can protect most vaccinated patients from the development of acute and chronic Hepatitis B. The vaccination of the newborn is currently the standard of care in most, even developing, countries. The long-term benefits of vaccination include the radical decrease of Hepatitis B morbidity but also the decrease of mortality resulting from decompensated cirrhosis or liver cancer [8].

Roma population migrated to Europe from North East India in the Middle Age [9]. Currently, most of the Slovakian Roma population lives in segregated settlements and, in comparison with non-Roma, has very different life-style, worse socio-economic circumstances and health care availability [10,11,12]. All these factors may influence the prevalence of Hepatitis B in Roma population of Slovakia. 

The aim of the presented work is to explore the prevalence of Hepatitis B and viremia in the region of East Slovakia with focus on the Roma people and compare various socio-economic variables and risk factors for Hepatitis B virus transmission between Roma and non-Roma populations.

## 2. Materials and Methods

Medium scale epidemiological research designed “HepaMeta” study was performed in the eastern Slovakia in 2011. It was designed as a cross-sectional population-based research. The general aim of the study was to explore prevalence of viral hepatitis B and C infection in association with wide spectrum of risk and protective factors in Roma population in east Slovakia and compare it to non-Roma population from the same region. Methodology overview follows, due to the extent of the study, detailed methodology for the study has been published elsewhere [13].

Ethics committee of Pavol Jozef Safarik University in Košice, Faculty of medicine approved the study protocol (no. 104/2011). The participation was voluntary and anonymous. Participants signed informed consent before the medical examination and sampling. The study was performed in compliance with the Declaration of Helsinki.

Because of high concentration of Roma population in the east Slovakia, the study was targeted to Košický region. The study was performed in cooperation with general practitioners (GP) working in this area. Nineteen GPs with Roma population in their coverage area were contacted and 12 agreed to participate (response 63%). Further seven randomly selected GPs, which provided care to mostly non-Roma population, were invited to participate. Roma people were invited to participate by local community workers. From all Roma people living in the selected settlements, 452 chose to participate. Because of unknown total population living in individual settlements and unpredictable conditions, we were not able to calculate response rate. Control non-Roma population was selected randomly from the databases of participating GPs. These people were contacted by telephone or mail or electronic mail, informed about the study and invited to participate. Out of 710 invited people 403 participated in the study (response rate 56.8%). All consenting participants were invited to their local cooperating GPs office, where the study samples, anthropometric measurements and medical information were collected by medical personnel. 

### 2.1. Measures

Questionnaires were administered to Roma people by research assistants; non-Roma participants had help with questionnaire on demand. Independent variables obtained through questionnaires included gender, ethnicity (Roma and non-Roma), previous incarcerations, drug use in general, i.v. drug use, sex for money or other reward, more than four sexual partners, tattoo in general, tattoo done privately, condom use always/most of the time, payment problems, household equipment and employment. “Payment problems” variable was an aggregate binary variable that contained the inability to pay one item of the following: rent, loan payment, healthcare, energies and other expenses. “Missing household equipment” was an aggregate binary variable that contained lacking at least one of the following sewage system, water supply, flush toilet, bathroom or shower, electricity supply. Highest obtained education level was categorised into Elementary, Middle and Higher education categories. Age and number of persons in living unit were expressed as interval variables. 

Medical history was obtained by supervising medical personnel from the general practitioners´ documentation. Relevant information included vaccination status against Hepatitis A or B, information about previously diagnosed common sexually transmitted diseases (Chlamydia, Gonorrhoea, Syphilis and genital Herpes) and blood transfusion all expressed as binary variables. 

Dependent variables were the positivity of HBsAg and antiHBcIgG and HBV DNA levels. Serology testing for HBsAg and antiHBcIgG was performed in all blood samples. HBV DNA was tested in HBsAg positive samples only. HBsAg and antiHBcIgG testing were performed by Enzygnost (Siemens, Eschborn, Germany); HBV DNA was measured by HBV Cobas Taqman Ampli Prep/Cobas v. 2.0 (Roche, Rotkreuz, Switzerland), with a detection limit of 20 IU/mL and an upper limit of 170,000,000 IU/mL. 

### 2.2. Definitions

Since the study was cross sectional and we did not test HBeAg positivity or antiHBcIgM positivity, we could not adhere to the canonical definitions as described in European Association for the Study of the Liver (EASL) guidelines from 2017 [14]. Instead we used the one-time HBsAg positivity as a surrogate marker of both chronic infection with Hepatitis B virus and chronic hepatitis B. Therefore, very few patients with acute Hepatitis B might be included (see Section 5. Strengths and limitations).

AntiHBcIgG positivity was considered to be a marker of overall lifetime contact with hepatitis B virus.

### 2.3. Statistical Analysis

Data is presented as absolute and relative counts with 95% confidence intervals for population in case of categorical data and mean with standard error of mean and 95% confidence intervals for population in case of interval data. First, we tested the difference (independence) of categorical variables by chi-squared test or Fisher exact test where appropriate and interval variables by *T*-test. Next, we calculated odds ratios by univariate logistic regression. Finally, we constructed fully adjusted multivariate logistic regression model that included all variables significant in the univariate regression.

## 3. Results

Altogether, 855 people participated in the study, of which 452 recruited from the Roma minority and 403 were from non-Roma population. Baseline parameters of the study cohort are summarized in the Table 1, which shows that, there are extreme differences in the demography, social, health and economic aspects of life between the two cohorts of participants. Test results for HBsAg were missing in 18 participants and antiHBcIgG in 19 participants. Global HBsAg positivity rate was 7.7% and antiHBcIgG positivity rate was 34.6%. HBV DNA positivity in HBsAg positive patients was 86.4%. The prevalence of HBsAg positivity was more than 4-times the prevalence of non-Roma population, similarly antiHBcIgG positivity. HBsAg positive Roma people were more commonly HBV DNA positive compared to HBsAg positive non-Roma population.

### 3.1. Hepatitis B surface Antigen Positivity

We used HBsAg positivity as a surrogate marker of chronic active Hepatitis B. There was significant difference in the prevalence of chronic Hepatitis B. More than 12% of Roma participants were HBsAg positive, compared to only 2.8% positive participants from the non-Roma population (*p* < 0.0001). Table 2 summarizes the risk factors for HBsAg positivity. 

Only significant risk factors for HBsAg positivity were Roma ethnicity, tattoo and economic factors such as problems with bill payments, unemployment and elementary education. Roma ethnicity conferred significantly higher odds (adjusted (OR) 4.556; 95% Confidence Interval (CI) 1.512–13.729; *p* = 0.007) of HBsAg positivity even after adjustment for age, sex, tattoo, payment problems, employment status and education. In the full regression model, only other significant predictor besides Roma ethnicity, was male sex (adjusted OR 1.821; 95% CI 1.023–3.242; *p* = 0.042) probably because high correlation between Roma ethnicity and lack of education, unemployment, poverty and tattoos, as seen in comparison of HBsAg positive Roma and non-Roma cohorts (Table 3). This comparison is limited by low number of HBsAg positives in the non-Roma, however the strong differences in the poverty and education remain, Roma people were more frequently tattooed but reported fewer sexual partners, lived in larger households, were more commonly unemployed and had only elementary education.

### 3.2. Hepatitis B Core Antigen Antibodies Positivity

AntiHBcIgG positivity was considered a marker of lifetime exposure to Hepatitis B virus. There was significant difference in the prevalence of antiHBcIgG positivity. More than half of Roma participants were in contact with Hepatitis B virus, compared to only about 16% of non-Roma population (*p* < 0.0001). Table 4 summarizes the risk factors for antiHBcIgG positivity.

Roma ethnicity along with elementary education carried the highest odds for antiHBcIgG positivity but most considered risk factors, except gender and sex for money were significantly different (i.v. drug application and a diagnose of sexually transmitted disease (STD) excluded due to very low number of participants). 

We constructed a multivariate regression model that included all significant risk factors from univariate analysis (fully adjusted model). The only independent and significant risk factors for antiHBcIgG positivity were Roma ethnicity (aOR 3.701; 95% CI 1.861–7.360; *p* < 0.0001), age (aOR 1.064; 95% CI 1.040–1.089; *p* < 0.0001) and elementary education (aOR 2.145; 95% CI 1.012–4.549; *p* = 0.047).

### 3.3. Hepatitis B Virus DNA Positivity

Out of 66 HBsAg positive patients, HBV DNA testing was performed in 63, out of which 57 participants were HBV DNA positive (86.4%). There was a marginally higher rate of HBV DNA positivity in Roma people compared to non-Roma (94.3% versus 70.0%; *p* = 0.046). HBsAg positive Roma people had higher odds of being also HBV DNA positive (OR 7.143; 95% CI 1.198–42.573; *p* = 0.031). Higher odds remained significant after the age adjustment (aOR 8.192; 95% CI 1.164–57.660; *p* = 0.035). Out of all other tested risk factors (same as for HBsAg and antiHBcIgG), only age of HBV DNA positive patients was marginally higher (mean 34.59 ± 0.90 versus 28.37 ± 2.97) but this difference did not reach statistical significance (*p* = 0.065).

### 3.4. Hepatitis B Virus DNA > 2000 IU/mL

We were also interested about the rate of HBV DNA above 2000 IU/mL, which is the recommended cut-off for the treatment initiation. Out of 57 HBV DNA positive patients 30 (52.6%) had HBV DNA over 2000 IU/mL. There was no difference in ethnicity (Roma versus non-Roma) between HBV DNA ≤ 2000 IU/mL and >2000 IU/mL (*p* = 0.238). There was also no statistically significant difference in all other analysed risk factors (same as for HBsAg and antiHBcIgG).

## 4. Discussion

The aim of this study is to map Hepatitis B prevalence in Roma and compare it to non-Roma population. We have found that people from Roma settlements in the eastern Slovakia have very high prevalence of Hepatitis B. When compared to non-Roma population, the rate of chronic Hepatitis B is significantly higher in Roma population living in segregated settlements compared to non-Roma population (12.4% versus 2.8%, *p* < 0.0001). Roma people from segregated settlements have significantly higher odds (aOR 4.556; 95% CI 1.512–13.729; *p* = 0.007) of HBsAg positivity even after adjustment for age, sex, tattoo, payment problems, employment status and education. Full regression model which included all significant risk factors from univariate analysis showed that only Roma ethnicity and male sex were independent and significant predictors of HBsAg positivity. This may be due to high correlation between Roma ethnicity and socio-economic variables found significant in the univariate analysis.

High prevalence of Hepatitis B has been reported mainly from developing countries [15]. In the developed countries, high prevalence’s have been described in the various minorities or migrant population. Hepatitis B prevalence in the general population in the USA is 0.3%, however the prevalence rises over 10% in migrants from South East Asia. This prevalence is very similar to the prevalence in the country of origin [16]. Hepatitis B prevalence in India varies from 2% to 8% [17]. It is significantly higher in tribal populations (11.9%) compared to non-tribal populations (3.1%) [18]. It is therefore no surprise that the Hepatitis B prevalence in all published studies was higher in Roma population, compared to non-Roma [2,19,20,21]. AntiHBc total or antiHBcIgG antibodies are positive in chronic Hepatitis B, however they also remain positive after an episode of acute Hepatitis B. The rate of antiHBcIgG positivity in this study was also significantly higher in Roma population compared to non-Roma (52.8% versus 15.9%; *p* < 0.0001).

We have found out that the lifestyle of Roma population living in segregated settlements included significantly higher rates of risk behaviour for direct blood transmission, specifically tattooing in general but also tattooing in private, drug use, blood transfusion and imprisonment (all *p* < 0.0001). Odd ratio for HBsAg positivity was significantly increased by tattoo in any setting (OR 1.885; 95% CI 1.099–3.235; *p* = 0.02) and tattoo done privately (OR 1.870; 95% CI 1.068–3.273; *p* = 0.026). When we compared only HBsAg positive patients, Roma people were more frequently tattooed in general and particularly in private (*p* = 0.013). Significant individual risk factors for antiHBcIgG positivity were: blood transfusion (OR 1.555; 95% CI 1.006–2.403; *p* = 0.046), no reported drug use (OR 0.995; 95% CI 0.992–0.998; *p* = 0.003), tattoo total (OR 2.81; 95% CI 2.023–3.904; *p* < 0.0001), tattoo privately (OR 3.288; 95% CI 2.319–4.662; *p* < 0.0001) and imprisonment (OR 3.908; 95% CI 2.106–7.253). Parenteral route of Hepatitis B virus transmission was confirmed by multiple studies. Hepatitis B is more common in prison setting compared to general population [22]. One meta-analysis showed that tattoo is a significant risk factor for hepatitis B virus transmission (OR 1.48; 95% CI 1.3–1.68), what was also confirmed in this study. The risk of transmission increased even more in patients with documented risky behaviour (OR 1.64; 95% CI 1.32–2.03) [23].

Sexual behaviour plays a principal role in Hepatitis B virus transmission. In our set of data, we observed that although Roma reported fewer case of high numbers of sexual partners (more than four) compared to non-Roma (10.3% versus 21,5%; *p* < 0.0001), they also less frequently reported using barrier method for contraception and prevention against STDs (9.3% versus 32.5%; *p* < 0.0001). There was no significant difference in the proportion of participants performing sex for any kind of reward or the prevalence of participants diagnosed with sexually transmitted disease between Roma and non-Roma. HBsAg positive Roma displayed different sexual behaviour. Although significantly larger proportion reported less than four sexual partners compared to non-Roma (13% versus 40%; *p* = 0.037), they also less frequently used condom as a barrier anticonception (20% versus 45.5%; *p* = 0.072). Anti HBcIgG positive patients used condom for protected sexual intercourse less commonly (OR 0.578; 95% CI 0.392–0.854; *p* = 0.005). Sexual transmission of Hepatitis B is very common but plays only sporadic role in Hepatitis C infection. On the other hand, transmission by blood is important in both Hepatitis B and C infection. Prevalence of Hepatitis C infection in Roma population is very small (0.7%) and is comparable to the prevalence in general population of Slovakia [2,24]. On the other hand, Hepatitis B prevalence is significantly higher in Roma population. Therefore, we can deduce that most of the horizontal transmission of Hepatitis B in Roma population occurs by sexual intercourse. In this study, the infrequent use of condom as a means for protected sexual intercourse was a significant risk factor for antiHBcIgG positivity. Bernabe-Ortiz et al. reported that the regular use of condom reduces the prevalence of antiHBcIgG antibodies threefold [25]. Synthetic condoms are superior to natural because of lower risk of leakage of Hepatitis B virus [26]. Described relationships support the necessity of sexual education of Roma people with the emphasis on the use of condom as a mean for protected sexual intercourse. 

In Roma population in segregated Roma settlements a significantly higher number of people living in one living unit (house or apartment) can be seen. In addition, this population more frequently reported problems with bills payment, lower education and employment rate and missing of common household equipment (all *p* < 0.0001). Worse education, lower employment rate and poverty were associated with HBsAg positivity. In addition, all of these variables with higher number of persons in a household and missing household equipment were associated with antiHBcIgG positivity. In every one of these parameters there was a significant difference between Roma and non-Roma HBsAg positive participants. These findings confirm the hypothesis that poverty and poor socio-economic status in general may be associated with higher prevalence of chronic Hepatitis B. In an older published study, no HBsAg positivity was documented in “gypsies” living in better socio-economic conditions compared to “ciganes,” living in greater poverty with 27% HBsAg positivity. AntiHBc total antibodies was also higher in “ciganes” compared to “gypsies” (72% versus 12%; *p* < 0.001) [27].

Regarding the risk of antiHBcIgG, fully adjusted model that contained all risk factors found significant in univariate analysis (Table 4) revealed that only Roma ethnicity (aOR 3.701; 95% CI 1.861–7.360; *p* < 0.0001), age (aOR 1.064; 95% CI 1.040–1.089; *p* < 0.0001) and elementary education (aOR 2.145; 95% CI 1.012–4.549; *p* = 0.047) were independent and significant risk factors for antiHBcIgG positivity.

Vaccination against Hepatitis B virus was sporadic in both Roma and non-Roma but more common in non-Roma (11.9% versus 3.3%; *p* < 0.0001). Vaccination against Hepatitis B is protective against the acquirement of the infection. Common universal vaccination of newborns against Hepatitis B virus was introduced in 1998. This study included only patients that were not vaccinated as newborns. Vaccination of adult population may stop the spreading of Hepatitis B and prevent fatal complications and thus be pharmacoeconomically effective, however in current situation not only medical but also societal support is necessary.

Roma people from segregated settlements have not only higher prevalence of Hepatitis B but also higher prevalence of active replication of infection in comparison to HBsAg positive non-Roma population participants (94.3% versus 70.0%; *p* = 0.046). Patients with confirmed HBV replication need to be followed-up regularly and significant proportion will require antiviral treatment in the future. Clinically significant viral replication (HBV DNA > 2000 IU/mL) had also the tendency to be more common in Roma population (56% versus 28.6%), however we were not able to confirm this statistically, due to low number of these participants. These patients in general require antiviral treatment. The availability of healthcare in segregated Roma settlements is worse compared to non-Roma [12]. Furthermore, also compliance to treatment is lower in Roma population. In a previously published retrospective analysis from our group we reported that Pegylated interferon alpha treatment was completed by 100% patients from the non-Roma but only 79% patients from Roma population (*p* = 0.0009). This led to the lower rate of virological response at the end of treatment in Roma population (51% versus 81%; *p* = 0.003) [28]. HBsAg positive participants in this study, who fulfilled treatment criteria, were contacted through their general practitioner and were offered the standard of care treatment with nucleot(s)ide analogues or pegylated interferon alpha. No patient received antiviral treatment prior to the inclusion to the study.

## 5. Strengths and Limitations

Main strength of this study is that it included Roma people from segregated settlements with difficult access to health care [29], furthermore the inclusion of patients was based on random sampling thus minimizing selection bias and decreasing the influence of potential confounders and the extent of gathered socio-economic variables. 

There are several limitations to this study, although none is critical to the scientific message. The data collection and sample analysis of the study was performed in 2011, however, there have been no significant changes in the health policies or participation of the Roma community in the health care. There also have not been significant changes in the socio-economic circumstances. Therefore, the prevalence data are as accurate as possible.

Because the study was cross-sectional, we did not test for antiHBcIgM and there was no follow-up testing we could have also included very low number of patients with acute Hepatitis B. However, the study included only adults and mean age was 34 years, therefore we believe that this is only a minor source of bias in this study.

## 6. Recommendations

Further research in this topic needs to be focused on the Hepatitis B prevention in the high-risk Roma population, including vaccination strategy. The results of this research may be reflected in social-care, including the education of Roma people about Hepatitis B risk factors, particularly the tattoo and piercing procedures and safe-sex practices. We also recommend the update for public health policies regarding vaccination against Hepatitis B in high risk Roma settlements and prioritization of antiviral treatment.

## 7. Conclusions

Chronic Hepatitis B prevalence is very high in Roma population in the east Slovakia. Every eight Roma is HBsAg positive, which is significantly higher compared to non-Roma. The prevalence of antiHBcIgG antibodies is also significantly higher in Roma. Roma ethnicity is an independent predictor of chronic Hepatitis B and the presence antiHBcIgG antibodies. Roma living in segregated settlements have higher rate of risk factors for parenteral (tattoo, blood transfusion, imprisonment and drug use) and sexual transmission (less frequent condom use) of Hepatitis B. Also, lifestyle and socio-economic conditions may be associated with Hepatitis B virus transmission. HBsAg positive Roma have more frequently also the HBV replication compared to HBsAg positive non-Roma. Universal vaccination of Roma population may decrease the rate of Hepatitis B transmission in this community and ameliorate the health-related and socio-economic consequences associated with chronic Hepatitis B. 

## Figures and Tables

**Table 1:** Characterisation of the study cohort according to ethnicity. SEM—Standard error of mean, CI—Confidence interval, I.v.—Intravenous.

	Roma (*n* = 452)			Non-Roma (*n* = 403)			*p* Value
	Count/Mean	(%)/SEM	95% CI	Count/Mean	(%)/SEM	95% CI	
Demography							
Male sex	159	35.2%	30.8%–39.8%	185	45.9%	40.9%–50.9%	0.001
Age (years)	34.67	0.26	33.72–35.46	33.51	0.37	32.48–34.03	0.043
Social							
Imprisonment	46	10.3%	7.74%–13.64%	4	1.0%	0.3%–2.7%	<0.0001
No reported drug use	315	69.7%	65.3%–73.6%	322	80.1%	75.9–83.7	<0.0001
I.v. drug application	2	0.5%	0.08%–1.8%	1	0.3%	0.01–1.7	ns
>4 sexual partners	44	10.3%	7.8%–13.5%	75	21.5%	17.5–26.1	<0.0001
Sex for Money	13	3.0%	1.7%–5%	8	2.2%	1.1–4.2	ns
Used condom always or most of the time	41	9.3%	7%–12.4%	117	32.5%	27.9–37.5	<0.0001
Tattoo total	173	39.1%	34.7%–43.8%	29	7.4%	5.2–10.5	<0.0001
Tattoo privately	164	37.1%	32.7%–41.7%	9	2.3%	1.2–4.3	<0.0001
Persons in living unit	7.44	0.26	6.94–7.96	4.27	0.22	3.83–4.71	<0.0001
Health related							
Hepatitis A vaccination	30	6.6%	4.7–9.3	33	8.2%	5.9–11.3	ns
Hepatitis B vaccination	15	3.3%	2.0–5.4	48	11.9%	9.1–15.4	<0.0001
Sexually transmitted disease	0	0.0%	0–1.0	2	0.5%	0.1–2.0	ns
Blood transfusion	71	16.7%	13.4%–20.5%	26	6.7%	4.6–9.7	<0.0001
Economic							
Payment problems **	218	48.2%	43.7%–52.8%	49	12.2%	9.3–15.7	<0.0001
Missing household equipment *	281	62.2%	57.6%–66.5%	78	19.4%	15.8–23.5	<0.0001
Employment	46	10.4%	7.9%–13.6%	284	73.6%	69–77.7	<0.0001
Elementary education	433	97.7%	95.9%–98.8%	93	23.7%	19.7–28.1	<0.0001
Middle education	10	2.3%	1.2%–4.1%	163	41.5%	36.–46.4	
Higher education	0	0.0%	0%–1.1%	137	34.9%	30.3–39.7	
Virology							
HBsAg positive	55	12.4%	9.58%–15.97%	11	2.8%	1.56%–4.91%	<0.0001
HBV DNA positive	50	94.3%	83.37%–98.53%	7	70.0%	35.37%–91.91%	0.046
antiHBcIgG positive	233	52.8%	48.17%–57.44%	63	15.9%	12.56%–20.02%	<0.0001
HBV DNA > 2000IU/mL	28	56.0%	42.31%–68.84%	2	28.6%	5.11%–69.74%	ns

ns—not significant; *—lacking at least one item of the following: sewage system, water supply, flash toilet. bathroom or shower, electricity supply; **—issue to pay at least one item of the following: rent, loan payment, healthcare, energies, other expenses.

**Table 2:** Risk factors for HBsAg positivity in the whole study population. SEM—Standard error of mean, CI—Confidence interval, OR—Odds ratio, I.v.—Intravenous.

	Negative (*n* = 773)		Positive (*n* = 64)		*p* Value	OR	95% CI
	Count/Mean	(%)/SEM	Count/Mean	(%)/SEM			
Demography							
Male sex	302	39.2%	34	51.5%	0.05	1.65	0.997–2.731
Age (years)	34.1	0.31	34.13	0.85	0.97	1	0.971–1.031
Category–Roma	387	50.2	55	83.3	<0.0001	4.961	2.558–9.624
Social							
Imprisonment	41	5.4%	7	10.6%	0.086	2.063	0.887–4.8
No reported drug use	578	75.1%	47	71.2%	ns	0.998	0.992–1.004
I.v. drug application	3	0.4%	0	0.0%	ns		
>4 sexual partners	103	14.8%	11	17.2%	ns	1.197	0.605–2.368
Sex for Money	19	2.6%	2	3.0%	ns	1.158	0.264–5.083
Used condom always or most of the time	138	19.2%	16	24.2%	ns	1.343	0.742–2.429
Tattoo total	176	23.4%	23	36.5%	0.02	1.885	1.099–3.235
Tattoo privately	150	19.9%	20	31.7%	0.026	1.870	1.068–3.273
Blood transfusion	86	11.7%	9	14.1%	ns	1.231	0.587–2.58
Sexually transmitted disease	2	0.3%	0	0.0%	ns		
Persons in living unit	6.01	0.2	6.78	3.58	ns	1.022	0.983–1.063
Economic							
Payment problems **	233	30.2%	29	43.9%	0.021	1.81	1.087–3.013
Missing household equipment *	318	41.2%	34	51.5%	ns	1.514	0.91–2.504
Employed	306	41.0%	16	24.6%	0.01	0.47	0.262–0.841
Elementary education	459	60.9%	58	89.2%	<0.0001	8.403	2.026–34.860
Middle education	162	21.5%	5	7.7%		2.052	0.392–10.749
Higher education	133	17.6%	2	3.1%		Refer	

ns—not significant *—lacking at least one item of the following: sewage system, water supply, flush toilet, bathroom or shower, electricity supply; **—issue to pay at least one item of the following: rent, loan payment, healthcare, energies, other expenses.

**Table 3:** Comparison of risk factors between HBsAg positive Roma and non-Roma population. SEM—Standard error of mean, CI—Confidence interval, OR—Odds ratio, I.v.—Intravenous.

	Roma (*n* = 55)			Non-Roma (*n* = 11)		95% CI	*p* Value
	Count/Mean	(%)/SEM	95% CI	Count/Mean	(%)/SEM		
Demography							
Male sex	28	50.9%	37.2–64.5	6	54.5%	24.6–81.9	ns
Age (years)	34.44	0.98	32.2–36.2	32.56	1.57	27.8–36.3	ns
Social							
Imprisonment	7	12.7%	5.7–25.1	0	0.0%	0–32.1	ns
No reported drug use	37	67.3%	54.1–78.2	10	90.9%	57.1–99.5	ns
I.v. drug application	53	0%	0–8.4	11	0.0%	0–32.1	ns
>4 sexual partners	7	13.0%	6.4–24.4	4	40.0%	13.7–72.6	0.037
Sex for Money	2	3.6%	0.6–13.6	0	0.0%	0–32.1	ns
Tattoo total	23	44.2%	31.6–57.7	0	0.0%	0–32.1	0.006
Tattoo privately	20	38.5%	26.5–52.0	0	0.0%	0–32.1	0.013
Blood transfusion	8	14.8%	7.1–27.7	1	10.0%	0.5–45.9	ns
Used condom always or most of the time	11	20%	11.6–32.4	5	45.5	18.1–75.4	0.072
Sexually transmitted disease	0	0.0%	0–8.1	0	0.0%	0–32.1	
Persons in living unit	7.27	0.49	6.3–8.2	3.63	0.75	1.8–5.4	0.006
Economic							
Payment problems **	26	47.3%	34.7–60.2	3	27.3%	7.3–60.7	ns
Missing household equipment *	31	56.4%	43.3–68.6	3	27.3%	7.3–60.7	0.078
Employed	7	12.7%	6.3–24.0	9	90.0%	54.1–99.5	<0.0001
Elementary education	53	98.1%	88.8–99.9	5	45.5%	18.1–75.4	<0.0001
Middle education	1	1.9%	0.1–11.2	4	36.4%	12.4–68.4	
Higher education	0	0.0%	0–8.3	2	18.2%	3.2–52.2	

ns—not significant; *—lacking at least one item of the following: sewage system, water supply, flash toilet. bathroom or shower, electricity supply; **—issue to pay at least one item of the following: rent, loan payment, healthcare, energies, other expenses.

**Table 4:** Risk factors for antiHBcIgG positivity. SEM—Standard error of mean, CI—Confidence interval, OR—Odds ratio, I.v.—Intravenous.

	Anti HBcIgG Positive (*n* = 289)		Anti HBcIgG Negative (*n* = 547)		*p* Value	OR	95% CI
	Count/Mean	(%)/SEM	Count/Mean	(%)/SEM			
Demography							
Male sex	120	40.5%	216	40.0%	ns	1.023	0.766–1.365
Category–Roma	233	78.7%	208	38.5%	<0.0001	5.903	4.253–8.194
Age (years)	36.59	0.46	32.7	0.36	<0.0001	1.059	1.040–1.078
Social							
Imprisonment	32	10.9%	16	3.0%	<0.0001	3.908	2.106–7.253
No reported drug use	204	68.9%	421	78.1%	0.003	0.995	0.992–0.998
I.v. drug application	1	0.4%	2	0.4%	ns	1.099	0.099–12.169
>4 sexual partners	33	11.9%	81	16.8%	0.071	0.671	0.435–1.037
Sex for Money	11	3.8%	10	2.0%	ns	1.946	0.816–4.640
Used condom always or most of the time	42	14.5%	112	22.7%	0.005	0.578	0.392–0.854
Tattoo total	107	37.0%	91	17.3%	<0.0001	2.810	2.023–3.904
Tattoo privately	98	33.9%	71	13.5%	<0.0001	3.288	2.319–4.662
Blood transfusion	42	14.9%	52	10.1%	0.046	1.555	1.006–2.403
Sexually transmitted disease	1	0.3%	1	0.2%	ns	1.827	0.114–29.317
Persons in living unit	6.61	0.22	5.76	0.26	0.028	1.035	1.002–1.070
Economic							
Payment problems **	131	44.3%	130	24.1%	<0.0001	2.504	1.850–3.389
Missing household equipment *	160	54.1%	191	35.4%	<0.0001	2.150	1.611–2.869
Employed	62	21.5%	260	49.8%	<0.0001	0.276	0.199–0.384
Elementary education	244	83.8%	272	51.6%	<0.0001	7.753	4.342–13.843
Middle education	33	11.3%	134	25.4%		2.128	1.087–4.167
Higher education	14	4.8%	121	23.0%		Refer	

ns—not significant; *—lacking at least one item of the following: sewage system, water supply, flush toilet, bathroom or shower, electricity supply; **—issue to pay at least one item of the following: rent, loan payment, healthcare, energies, other expenses.
